# Yeast glucose pathways converge on the transcriptional regulation of trehalose biosynthesis

**DOI:** 10.1186/1471-2164-13-239

**Published:** 2012-06-14

**Authors:** Eva Apweiler, Katrin Sameith, Thanasis Margaritis, Nathalie Brabers, Loes van de Pasch, Linda V Bakker, Dik van Leenen, Frank CP Holstege, Patrick Kemmeren

**Affiliations:** 1 Molecular Cancer Research, University Medical Centre Utrecht, Universiteitsweg 100, Utrecht, the Netherlands; 2 Netherlands Bioinformatics Centre, Geert Grooteplein 28, 6525, GA, Nijmegen, the Netherlands

**Keywords:** Regulatory networks, Glucose signalling, Trehalose biosynthesis, Gene expression profiling, *Saccharomyces cerevisiae*

## Abstract

**Background:**

Cellular glucose availability is crucial for the functioning of most biological processes. Our understanding of the glucose regulatory system has been greatly advanced by studying the model organism *Saccharomyces cerevisiae*, but many aspects of this system remain elusive. To understand the organisation of the glucose regulatory system, we analysed 91 deletion mutants of the different glucose signalling and metabolic pathways in *Saccharomyces cerevisiae* using DNA microarrays.

**Results:**

In general, the mutations do not induce pathway-specific transcriptional responses. Instead, one main transcriptional response is discerned, which varies in direction to mimic either a high or a low glucose response. Detailed analysis uncovers established and new relationships within and between individual pathways and their members. In contrast to signalling components, metabolic components of the glucose regulatory system are transcriptionally more frequently affected. A new network approach is applied that exposes the hierarchical organisation of the glucose regulatory system.

**Conclusions:**

The tight interconnection between the different pathways of the glucose regulatory system is reflected by the main transcriptional response observed. Tps2 and Tsl1, two enzymes involved in the biosynthesis of the storage carbohydrate trehalose, are predicted to be the most downstream transcriptional components. Epistasis analysis of *tps2*Δ double mutants supports this prediction. Although based on transcriptional changes only, these results suggest that all changes in perceived glucose levels ultimately lead to a shift in trehalose biosynthesis.

## Background

**Figure 1:**
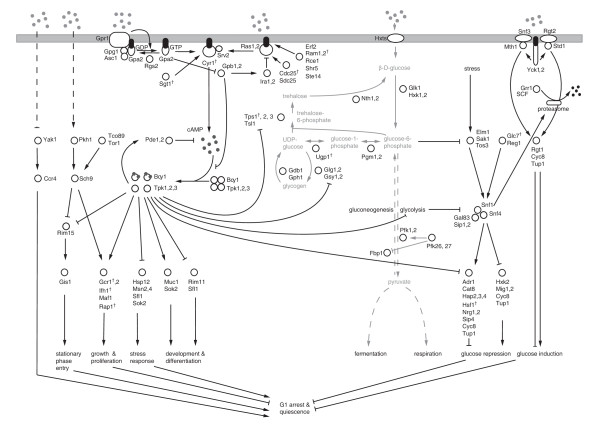
**An overview of the yeast glucose signalling and metabolic pathways.** Signalling events are indicated in solid black, metabolic reactions in solid grey lines. Dashed black lines imply glucose signals activating the signalling components, dashed grey lines summarise metabolic reactions, e.g. glycolysis and gluconeogenesis. The metabolic pathways synthesising the storage carbohydrates glycogen and trehalose are depicted in detail. ^†^ indicates that the deletion mutant is lethal.

Many organisms have evolved survival strategies centred on glucose as their chief cellular carbon and energy source. Cellular glucose availability governs most biological processes such as growth, division, metabolism and the ability to deal with environmental stresses. Our understanding of glucose signalling in eukaryotes has been greatly advanced by studying the model organism *Saccharomyces cerevisiae*. Despite its relative simplicity, yeast has developed a complex system to monitor external glucose levels and faithfully relay this information to adjust metabolic and gene expression programmes accordingly. There are in fact several distinct upstream regulatory pathways for glucose regulation, including the Ras/PKA, Gpr1/PKA, Sch9, Yak1, Snf1 and Snf3/Rgt2 signalling pathways, as well as the metabolic pathways (Figure 1; for comprehensive reviews, see [1,2]). Although transmission of the glucose signal is thought to be redundant [1,3], each pathway possesses distinct glucose detection and signal transmission methods.

The Protein Kinase A (PKA) pathway, which is regulated upstream by Ras and Gpr1, is pivotal for the glucose response. In periods of high glucose abundance, it directs the cell to ferment the available glucose to support growth and proliferation, whilst simultaneously repressing the stress response and the use of alternative carbon sources. Ras1 and Ras2 are small monomeric GTPases. In response to high glucose levels, Ras1 and Ras2 are activated and bind to adenylate cyclase, which is composed of Cyr1 and an associated protein Srv2 [4,5]. The subsequent increase in cyclic AMP (cAMP) production activates PKA. A GPCR system operates in parallel to Ras [5-8]. Upon sensing high glucose levels, Gpr1, the 7-transmembrane receptor, accelerates the GDP for GTP exchange on the Gα-subunit Gpa2, which then activates Cyr1 and thus raises PKA activity. The Sch9 pathway operates in parallel to PKA to couple glucose availability and growth by regulating ribosomal biogenesis and ribosomal protein transcript levels [3,9]. The Yak1 and Snf1 signalling pathways are triggered upon depletion of external glucose levels. The protein kinase Yak1 phosphorylates Pop2, part of the Ccr4-Not complex, to regulate transcript levels of stress response and carbohydrate metabolism genes [10] in a manner antagonistic to PKA [11]. The kinase Snf1 orchestrates the adaption yeast undergoes upon glucose depletion by mediating derepression of glucose-repressed genes and contributes to the response to other environmental stresses [12,13]. Last, the Snf3/Rgt2 signalling pathway consists of the extracellular glucose sensors Snf3 and Rgt2 that modulate the expression of numerous sugar transporter genes (the Hxts, Gal2, Stl1 and Agt1) [14,15].

While the yeast glucose regulatory system has been intensely investigated for decades, with many components and their relationships well defined, numerous aspects remain elusive. Examples include the precise characterisation of connections between the different pathways, determination of the hierarchical organisation of these pathways, as well as establishing the exact contribution of individual components to the overall glucose regulatory system.

Most components of the glucose regulatory system have been assigned to pathways based on a measurable phenotype caused by perturbation of that particular pathway. A classic example is the genetic screen using the sucrose non-fermenting phenotype of yeast mutants, which revealed various components, such as the Snf1 kinase, to be involved in glucose repression [16]. However, such phenotypes are often specific for individual pathways and hinder systematic comparison of a large number of components from different pathways side by side. Changes at the transcript level underlie many phenotypes. If measured collectively, for example by DNA microarray analysis of deletion mutants, such gene expression profiles can be exploited as detailed molecular phenotypes to systematically characterise many different pathways simultaneously using a single assay [17,18]. Similar approaches have previously been applied to analyse the yeast glucose regulatory system [3,5], but these studies have been limited to analyses of only a few components. In addition, the use of different strain backgrounds and experimental conditions hinders a systematic comparison between datasets. Here, DNA microarray gene expression profiles of deletion mutants are generated under a standardised high glucose growth condition to obtain a comprehensive overview of the yeast glucose regulatory system. In addition to relating gene expression profiles of pathway members by their similarity, the data is used to link cause and effect by relating the deleted gene to all transcripts significantly changing in response to the deletion [19,20]. To fully exploit the data, a new approach is devised that combines both these strategies to infer the underlying transcriptional regulatory network.

Here, we show that the pathways involved in glucose signalling are so tightly interlinked that in effect only one main transcriptional response can be discerned upon disruption of any individual pathway. This response varies in direction to mimic either a high or a low glucose response and reveals both known and unknown relationships within and between individual pathways and their members. In addition, a new network approach uncovers regulatory processes underlying the observed gene expression profiles. The results indicate that pathway members involved in the biosynthesis of the storage carbohydrate trehalose, Tps2 and Tsl1, are the most downstream transcriptional components. The study provides evidence that in response to a perceived alteration in external glucose levels the availability of the storage carbohydrates glycogen and trehalose is regulated, indicative of a shift in the metabolic programme.

## Results

### Gene expression profiles of the glucose regulatory system

For a better understanding of the glucose regulatory system as a whole, it is important to discern how individual pathway members of the system relate to each other. To systematically investigate these relationships, gene expression profiles were generated for 91 deletion mutants under a single condition (Synthetic Complete medium (SC), supplemented with 2% glucose). The mutant strains comprised all the non-essential genes implicated in the glucose regulatory system and include members of the Ras/PKA, Gpr1/PKA, Sch9, Yak1, Snf1 and Snf3/Rgt2 pathways, as well as rate-limiting metabolic enzymes (Figure 1; Additional file 1 and Additional file 2).

Each strain was profiled four times from two independent cultures. Wildtype (WT) cultures (56 in total) were grown and profiled alongside sets of deletion mutants on each day to control for biological and technical variation. Statistical modelling results in an average gene expression profile that consists of *p* values and changes in mRNA expression for each gene, relative to the expression in an additional collection of 200 WT cultures [18]. The number of gene expression changes in the individual mutants varies considerably (Supplementary Figure 1 in Additional file 3), but none of the 56 WT gene expression profiles generated in parallel exhibit twelve or more genes changing significantly (*p* < 0.01, fold-change (FC) > 1.7). Applying the same threshold on the individual mutants, 51% (46) behave like WT and 49% (45) show changes in their gene expression relative to WT. Predictably, many mutants that behave like WT are known not to be required for, or are actively repressed under the condition investigated here. For instance, the hexokinases Hxk1 and Glk1 are subject to glucose-induced repression [21], so that under the condition investigated here their deletion bears no consequence. In other instances, redundancy might play a role, such as for the transcriptional regulators Nrg1 and Nrg2, which have overlapping functions [22].

### Deletion mutants mimic either a high or a low glucose response

**Figure 2:**
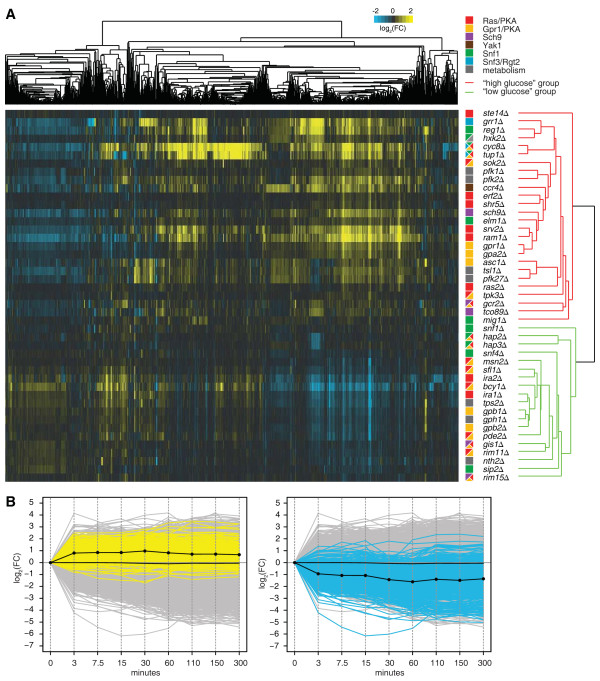
**Transcriptional response mimicking either a high or a low glucose response.** (**A**) Unsupervised hierarchical cluster diagram of all deletion mutants with gene expression changes differing from WT, i.e. twelve or more significant transcriptional changes, and all transcripts changing significantly in at least one of these mutants (*p* < 0.01, FC > 1.7). The dendrograms indicate relationships between transcripts (top) and mutants (right). The latter is colour-coded according to whether the mutants are part of the “high glucose” (red) or “low glucose” group (green). FC is indicated by the colour scale, with yellow for upregulation, blue for downregulation, and black for no change, versus the average WT. (**B**) Line graph of a time-course experiment in which glucose-depleted WT cells were inoculated into fresh media (SC, supplemented with 2% glucose) and their subsequent transcriptional output was monitored over a period of five hours. All transcripts differentially expressed between the “high glucose” and “low glucose” groups were split according to whether they were up- (left panel, yellow) or downregulated (right panel, blue) in the “low glucose” group. The average expression of the differentially expressed transcripts is indicated in black; all other transcripts are shown in grey.

The relationships between the 45 mutants with significant gene expression changes were investigated by hierarchical clustering of the gene expression profiles (Figure 2A). Gene expression profiles of deletion mutants can be treated as detailed molecular phenotypes [17,18]. Deleting certain pathway members often results in the malfunctioning of the entire pathway, the effect of which can be a specific expression signature. Deletion mutants of the same pathway will therefore show the same expression signature. Deletion mutants of distinct pathways, such as the HOG or mating pathway [18], or chromatin interaction pathways [23], show an expression signature specific to the pathway they belong to. The glucose regulatory system is composed of the Ras/PKA, Gpr1/PKA, Sch9, Yak1, Snf1 and Snf3/Rgt2 signalling pathways, as well as metabolic pathways (Figure 1). Nevertheless, based on the hierarchical clustering, the mutants segregate into two distinct groups rather than according to specific pathway membership (Figure 2A). Essentially, the expression signature of all members within one group is highly similar and mostly opposite to that of the other group, indicating that the two expression signatures are mutually exclusive. Thus, disruption of any glucose pathway causes an invariable response differing only in terms of direction and magnitude. A likely interpretation is that the pathways are so tightly interconnected that upon perceived alterations to glucose levels, they ultimately end up in one of two possible steady-states.

One aspect unifying mutants within each group is that members of one group, for instance Bcy1, Ira1/2 and Pde2, promote processes required under low glucose conditions (Figure 2A, “low glucose” group). In contrast, members of the other group, such as Gpr1, Ras2 and Reg1, promote processes required under high glucose conditions (Figure 2A, “high glucose” group). To determine whether the transcript changes elicited in the deletion mutants directly relate to the yeast glucose response, a time-course was conducted. Glucose-depleted WT cells were inoculated into fresh media (SC, supplemented with 2% glucose) and their subsequent transcriptional output was monitored over a period of five hours (Figure 2B; see Methods). All transcripts differentially expressed between the two groups of deletion mutants were split according to whether they were up- (Figure 2A, left) or downregulated (Figure 2A, right) in the “low glucose” group (see Methods; Additional file 4). Importantly, transcripts upregulated in deletion mutants of the “low glucose” group are also upregulated in WT cells upon the addition of glucose (Figure 2B, left panel). These transcripts are mainly involved in translation, for example the GeneOntology (GO; [24]) biological process “ribosome biogenesis” (*p* = 3.45E-45; see Methods). Likewise, transcripts downregulated in deletion mutants of the “low glucose” group are also downregulated in WT cells upon glucose addition (Figure 2B, right panel). These transcripts are enriched for “oxidation reduction process” (*p* = 2.47E-19), “trehalose metabolic process” (*p* = 5.80E-8), “cellular respiration” (*p* = 7.27E-7) and various metabolism related processes (see Additional file 4 for a full list of GO categories). Simultaneous repression of transcripts involved in respiration and induction of transcripts involved in translation are hallmarks of a high glucose response. By coupling fermentative growth to increased protein production, maximal growth rates are achieved. In contrast, deleting members of the “high glucose” group results in a low glucose response. Transcripts upregulated in this group are downregulated in WT cells upon the addition of glucose. Taken together, this strongly suggests that the gene expression profiles of mutants of the glucose regulatory system components are truly characteristic of a WT cell encountering either high or low glucose conditions. Moreover, it also supports the previously made observation that the cell shifts its metabolic and transcriptional programme based on the perceived rather than the actual glucose conditions [3,25-28].

In addition to the transcripts oppositely regulated across the two groups, other transcripts are affected in a mutant- rather than in a group- or pathway-specific way and reflect additional roles other than in glucose signalling (Figure 2A; Additional file 4). Two examples include *tup1Δ* and *cyc8Δ* that show many specific transcript changes. This agrees with the fact that the Tup1-Cyc8 general co-repressor complex is also known to directly repress genes involved in functions as diverse as DNA damage, mating, oxygen response [29] and amino acid metabolism [30].

### Gene expression profiles expose relationships between components of the glucose regulatory system

Although each deletion mutant globally falls either into the “high glucose” or “low glucose” group, the gene expression profiles within each group still show different degrees of similarity (Figure 2A). Within each group, the gene expression profiles are organised in a manner largely consistent with the current understanding of the yeast glucose regulatory system. For instance, gene expression profiles of members of the same protein complex such as *gpr1Δ* and *gpa2Δ*[1,28] or the palmitoyltransferase subunits *erf2Δ* and *shr5Δ*[31], cluster tightly. Similarly, deletions of homologous components, such as Gpb1 and Gpb2 [28], also result in highly similar gene expression profiles. Likewise, cooperating members cluster tightly together, e.g. *grr1Δ, reg1Δ, hxk2Δ, cyc8Δ* and *tup1Δ*, which collectively mediate glucose repression [32-35]. This indicates that although a great proportion of transcripts are involved in the high or low glucose response, more subtle relationships can still be detected through the transcriptional response of these mutants.

**Figure 3:**
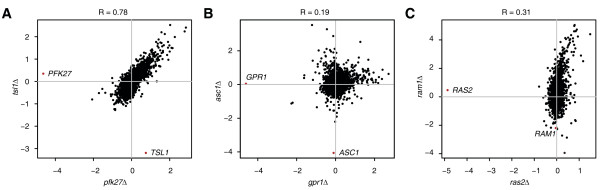
**Relationships exposed through gene expression profiling.** Transcript changes (FC) of two different deletion mutants are plotted against each other. Red dots indicate the deleted genes. (**A**) Transcript changes of the *pfk27Δ* and *tsl1Δ* mutants are highly correlated. (**B**) Transcriptional changes of the *asc1Δ* mutant are not negatively correlated to those of the *gpr1Δ* mutant suggesting that Asc1 does not inhibit Gpr1. (**C**) The deletion of *RAM1* results in many more transcriptional changes than the deletion of *RAS2*. *RAS1* is not shown as its deletion behaves like WT.

In addition to established relationships such as those described above, a number of previously uncharacterised relationships can be inferred from the gene expression profiles. The tight correlation observed between the gene expression profile of *tsl1Δ* and *pfk27Δ* (Figure 3A) is indicative of a functional relationship. This is further substantiated by their positive genetic interaction as derived from a high-throughput synthetic genetic interaction map [36], which can signify that both gene products are part of the same complex or pathway. Until now, no concrete role has been assigned to Tsl1 but it is speculated to have regulatory functions within the trehalose synthase complex [37]. Pfk27 is the 6-phosphofructo-2-kinase that synthesises the key metabolite fructose-2,6-bisphosphate, which regulates the glycolytic/gluconeogenic switch. The correlation between the *tsl1Δ* and *pfk27Δ* gene expression profiles indicates a regulatory link between storage carbohydrate synthesis and the shift from glycolysis to gluconeogenesis and vice versa.

The identity of the β-subunit of the glucose sensing G-protein of the Gpr1/PKA pathway is much debated [38]. Previously, Asc1 has been proposed to fulfil this role [39]. Should Asc1 inhibit Gpr1 signalling, its gene expression profile would be the inverse of that of *gpr1Δ*. Remarkably, this is not observed (Figure 2A; Figure 3B). As evident from Figure 2A, the *asc1Δ* gene expression profile clusters closely with that of *pfk27Δ* and *tsl1Δ*, strongly indicating that Asc1 is not the β-subunit of the Gpr1 system but instead shares a functional role with Pfk27 and Tsl1 in storage carbohydrate synthesis and the glycolytic/gluconeogenic switch.

Another interesting new putative functional relationship concerns Ram1. Ram1 is the β-subunit of the CAAX farnesyltransferase [40], which prenylates Ras1, Ras2 and the a-factor mating pheromone to tether them to the membrane. The extensive transcriptional changes elicited by its deletion imply that Ram1 plays a much more important role than previously thought (Figure 2A; Figure 3C). This can be explained by the fact that in a *ram1Δ* strain, Ras1 and Ras2 are mislocalised to the cytosol and presumably forfeit their signalling capacity [41]. Ram1 should therefore be accredited with a major role in the Ras/PKA branch of the glucose regulatory system, rather than being thought of as a supporting actor. These examples demonstrate that using gene expression profiles as detailed molecular phenotypes can reveal many different types of functional relationships.

### Metabolic pathway members are transcriptionally regulated

**Figure 4:**
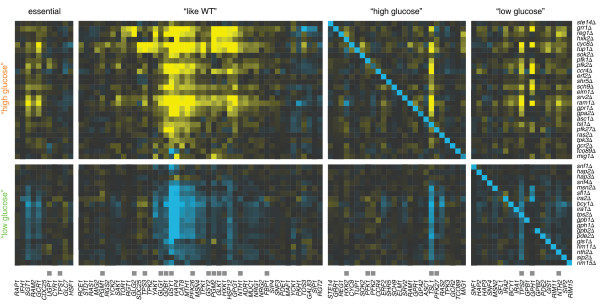
**Transcriptional regulation within the glucose regulatory system.** Transcript changes (horizontal) of all essential pathway members, those that upon deletion still behave like WT (“like WT”), as well as transcripts of pathway members categorised into the “high glucose” and “low glucose” group are depicted in the different mutants (vertical). Grey boxes indicate pathway members involved in the metabolic pathways. Colour scale and order of mutants (vertical) as in Figure 2. Transcripts (horizontal) of essential pathway members and members corresponding to mutants that behave like WT are ordered as derived from the hierarchical clustering. The transcript ordering of pathway members included in the “high glucose” and “low glucose group” is the same as for the mutants. The diagonal depicts the deleted genes.

The main transcriptional response (Figure 2) indicates a tight interconnection between the individual pathways of the glucose regulatory system. To investigate the degree to which components of the glucose regulatory system transcriptionally influence each other, the effect of deleting one pathway member on the mRNA expression of all the other pathway members was systematically determined (Figure 4). When assayed in this way, members of the Ras/PKA, Gpr1/PKA, Sch9, Yak1, Snf1 and Snf3/Rgt2 signalling pathways are only infrequently regulated at the mRNA level. In contrast, genes whose transcription is frequently changed encode members of the metabolic pathways (Figure 4, indicated by grey boxes; Supplementary Figure 2 in Additional file 3), especially enzymes involved in the biosynthesis of glycogen and trehalose. Their transcript levels are strongly increased in deletion mutants of the “high glucose” group (Figure 4, top) and decreased in deletion mutants of the “low glucose” group (Figure 4, bottom). With the exception of Gph1, Nth2, Tps2 and Tsl1, metabolic pathway members regulated at the level of transcription do not result in significant transcriptional changes upon their own deletion, most likely because their activity is not required under the high glucose conditions used in this study. Taken together, these analyses indicate that changes in perceived glucose levels ultimately lead to a shift in the metabolic programme, either to or from fermentation, and that this is achieved by regulating the transcription of metabolic pathway members, such as Gsy1, Gdb1, Tps2 and Tsl1.

### Tps2 is the most downstream transcriptional component

**Figure 5:**
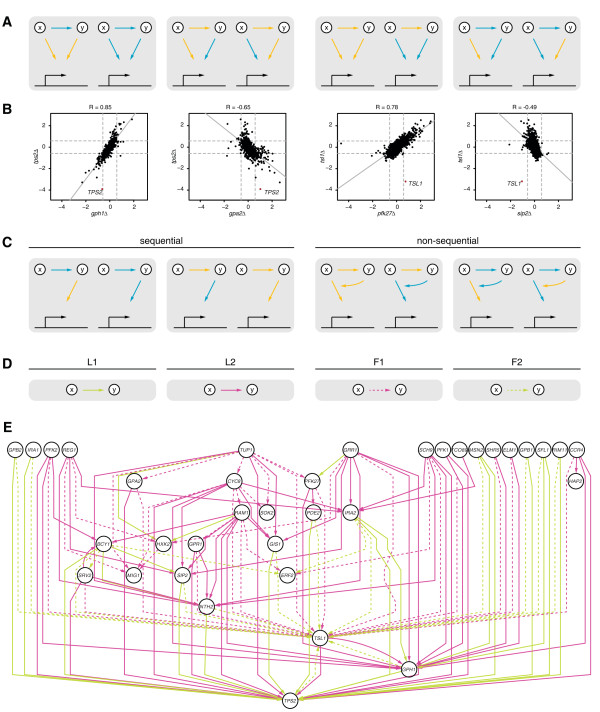
**Hierarchical network reconstruction.** Tps2 is the most downstream transcriptional component. (**A**) Possible data observations. A blue edge from *x* to *y* indicates decreased transcription of *y* in the deletion of *x*, a yellow edge indicates increased transcription. Edges of the same colour going from *x* and *y* to downstream target genes denote correlation between the gene expression profiles of *xΔ* and *yΔ*, anti-correlation otherwise. (**B**) Different types of data observations, as presented in (A), are exemplified. Dashed grey lines indicate 1.5 FC. Solid grey lines indicate the linear regression line fitted through the data points. Deletions of *x* and *y* are represented on the x- and y-axis respectively. The transcriptional change of *y* is highlighted by a red dot. (**C**) Data interpretations. The two leftmost types of data observations are interpreted as sequential relationships, in which transcriptional changes of downstream target genes observed in the deletion of *x* are indirect through the transcriptional regulation of *y*. The two rightmost types of data observations are interpreted as non-sequential relationships such as feedback from *y* to *x* itself or downstream target genes of *x*. (**D**) Unique edges (green and purple, solid and dashed) are used to denote the different types (L1, L2, F1 and F2, see Methods for details) of data observations in the network. (**E**) Data observations are summarised and represented as described in (D) for all components of the glucose regulatory system that upon deletion result in significant transcriptional changes compared to WT.

To further determine whether members of the metabolic pathways are indeed the most downstream transcriptional components, a new approach was applied to deduce the hierarchy of transcriptional regulation within the glucose regulatory system (see Methods). The approach is designed to explain a gene expression profile measured upon the deletion of one pathway member through the transcriptional regulation of another pathway member. Two measures are used to define the hierarchical relationship between two pathway members: (a) the transcript change they elicit on each other, and (b) the correlation of their gene expression profiles. Depending on the sign of these measures, four possible combinations are distinguished (Figure 5A, B) and categorised into two types (Figure 5C) “sequential” and “non-sequential”. A sequential relationship is observed when the transcript changes for a pathway member as a result of its deletion can be explained by the altered transcription of a second pathway member (Figure 5A-D, left). This is the case, for instance, when the transcript level of pathway member *y* is reduced upon the deletion of pathway member *x*, resulting in a gene expression profile highly similar to the deletion of *y* itself. In the reconstructed transcription network, pathway member *y* is hence placed downstream to pathway member *x*. On the other hand, gene expression profiles may indicate a non-sequential relationship, such as a feedback circuit between two pathway members (Figure 5A-D, right). In this case, the transcriptional regulation of one pathway member cannot easily explain the gene expression profile of the second pathway member, indicating a non-sequential relationship that involves additional intermediate components.

The combination of all such relationships found between components of the glucose regulatory system is depicted in a hierarchical network (Figure 5E). Interestingly, components are typically either found in sequential relationships, e.g. Bcy1, Gph1, or Tps2, or are predicted to be involved in non-sequential relationships such as feedback, e.g. Erf2, or Tsl1. Consistent with the previous analysis (Figure 4), the network shows that metabolic pathway members involved in trehalose biosynthesis, in particular Tps2 and Tsl1, are the most downstream transcriptional components of the glucose regulatory system and are therefore predicted to mediate the main transcriptional response to perceived glucose availability (Figure 2).

**Figure 6:**
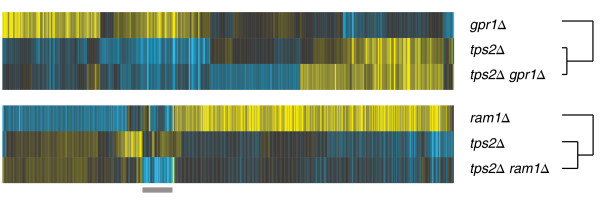
**Tps2 is epistatic to both Gpr1 and Ram1.** Transcriptional changes upon the single deletion of either *TPS2* or *GPR1*, as well as *TPS2* or *RAM1* are compared to the effect of their combined deletion. Shown are all transcripts (horizontal) changing significantly (*p* < 0.01, FC > 1.7) in any of the three deletion mutants (vertical). In both *tps2Δgpr1Δ* and *tps2Δram1Δ* double deletions, transcriptional changes of *tps2Δ* dominate the double mutant gene expression profile. Colour scale as in Figure 2.

**Figure 7:**
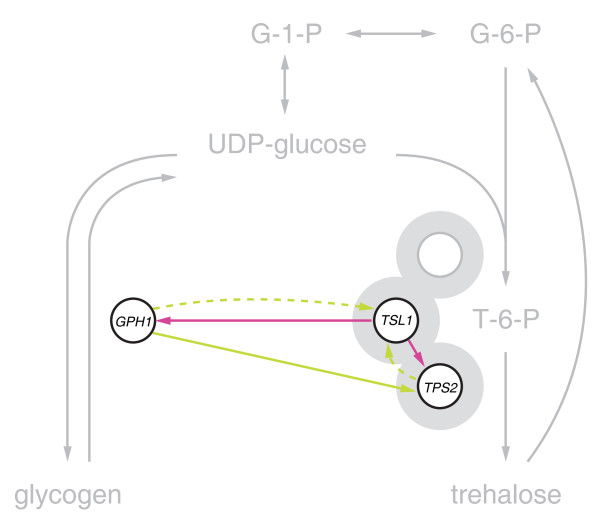
**A regulatory unit for balancing the storage carbohydrate biosynthesis.** The inferred transcription network between Tps2, Tsl1, and Gph1 (purple and green; as in Figure 5E) is integrated into the metabolic pathway (grey) in which they are functioning.

Trehalose is synthesised by a complex consisting of four members: the trehalose-6-phosphate synthase Tps1, the trehalose-6-phosphate phosphatase Tps2, as well as the regulatory subunits Tsl1 and Tps3. Tps1 is essential for growth on rapid fermentative carbon source as used in this study, and therefore a gene expression profile of *tps1Δ* could not be determined. Of the remaining complex members only deletion of either Tps2 or Tsl1 leads to significant transcript changes, suggesting that Tps3 is not required for the functioning of the complex under high glucose conditions. The transcriptional regulation of Tps2 may account for the global transcriptional changes measured upon the deletion of various components of the glucose regulatory system. To further investigate this prediction, we performed epistasis analysis by gene expression profiling double mutants. These mutants consisted of *tps2Δ* in combination with the deletion of *GPR1* and *RAM1*, two members of the Gpr1/PKA and Ras/PKA pathways that have a gene expression profile opposite to *tps2Δ*. Epistasis can describe a genetic interaction between two genes, in which the deletion of one gene masks or suppresses the effects of the other gene [42]. Tps2 is then epistatic to and in fact acting downstream of Gpr1 and Ram1 if the gene expression profile of the respective double mutant resembles the profile of the *tps2Δ* single mutant. Gpr1 indeed functions upstream of Tps2 as reflected in the gene expression profile of the *tps2Δ gpr1Δ* double mutant, which is most similar to the *tps2Δ* profile and the inverse of the *gpr1Δ* profile (Figure 6, top). Similarly, based on the transcriptional hierarchy, Ram1 would be placed upstream of Tps2, in agreement with its role in membrane anchoring of the Ras proteins. The validity of this prediction is shown by the *tps2Δ ram1Δ* double mutant, which is again most similar to the *tps2Δ* gene expression profile (Figure 6, bottom). One exception is a set of genes enriched for the GO biological process “response to pheromone” (*p =* 8.50E-13), which can be accounted for by Ram1 being known to also prenylate the a-factor mating pheromone (Figure 6, grey bar) [40]. The decreased transcription of these genes are the only remainder of the *ram1Δ* single mutant that is retained in the *tps2Δ ram1Δ* double mutant gene expression profile and appears to be mediated independently of Tps2. While the precise function of Tsl1 is largely unknown, the network analysis suggests that it plays an important role in communicating a feedback signal to other components of the glucose regulatory system (Figure 5E). The balance between glycogen mobilisation and trehalose biosynthesis in particular is predicted to be mediated by Tsl1 through feedback (Figure 7) as further discussed below.

## Discussion

### Parallel pathways in the glucose regulatory system

Previous studies have exposed the existence of parallel pathways in the glucose regulatory system by showing that there still is a response to altered glucose levels in deletion mutants of individual glucose signalling pathways [3,5]. Here, we show by gene expression profiling all non-essential components of the glucose regulatory system that, in general, removing any single component of this system results in one main transcriptional response (Figure 2). Those pathway members that hardly show any transcriptional changes as a result of the deletion mutant are either not required under the condition investigated here or are known to have redundant partners within a given pathway. The loss of one pathway member can then be buffered by the existence of another and hence, does not lead to great transcriptional changes. Remarkably, for most deletion mutants of different pathways of the glucose regulatory system this is not the case, implying that these pathways do not buffer each other’s activity. This seems to suggest that the degree of genetic redundancy between pathways of the glucose regulatory system is negligible. Rather, they represent parallel pathways that are so interconnected that the ultimate transcriptional change measured upon their disruption is highly similar.

### Transcriptional response according to perceived glucose levels

One of the most striking observations is that, in general, the transcriptional response as a result of the deletion of a gene involved in the glucose regulatory system is invariably of the same type, although the magnitude and direction of the response vary (Figure 2A). Two groups of pathway members can be distinguished: those promoting processes required under low glucose conditions (“low glucose” group), and those promoting processes required under high glucose conditions (“high glucose” group). Transcripts differentially expressed between deletion mutants of these two groups are also involved in the physiological response to altered glucose levels (Figure 2B). However, since there is no actual difference in external glucose levels, the transcriptional response is based solely on perceived rather than actual external glucose levels, as has been suggested before [3,25-28].

### Uniform gene expression profiling to relate pathway members

Since standardised, uniform conditions are used for all experiments, different components of the glucose regulatory system can be related by the similarity of their gene expression profile. In this case, the gene expression profiles are used as detailed molecular phenotypes, rather than using them as a screen for finding differentially expressed genes. For example, we detect the well-established functional relationship between Gpr1 and Gpa2 and reveal unknown ones, such as between Tsl1, involved in trehalose metabolism, and Pfk27, involved in the regulation of glycolysis (Figure 3A). This seems to suggest that Tsl1 has a role in glycolytic regulation or, alternatively, might couple regulation of trehalose metabolism to the glycolytic flux. The concept of trehalose metabolism and the glycolytic flux being interdependent is very appealing since this allows the cell to couple its storage carbohydrate levels to the current metabolic rate and thus glucose availability.

### Transcriptional regulation of the glucose regulatory system

This study further investigated the extent to which the glucose regulatory system itself is regulated through transcription. Pathway members involved in signalling are hardly transcriptionally affected in the deletion mutants. It seems highly unlikely that they are not regulated at all, suggesting that they are regulated post-translationally to evoke changes in the signalling output at a faster rate. Our results indicate that the glucose levels perceived by the cells are propagated downstream through the signalling pathways to adjust the long-term metabolic output accordingly. In particular, the enzymes involved in the biosynthesis of the storage carbohydrates glycogen and trehalose are transcriptionally changed (Figure 4). This is supported by the fact that only upon gradual depletion of external glucose, cells begin to synthesise storage carbohydrates and therefore require the presence and transcriptional activation of the corresponding metabolic enzymes.

A new network approach is introduced, which is set up to reveal hierarchy and feedback in the observed transcriptional responses using the unique characteristics of deletion mutant gene expression profiles. The relation between two respective pathway members is explained by combining the similarity in their gene expression profiles with the effect that deletion of one pathway member has on the transcription of the other member. In fact, the approach can be applied to any system or pathway where determination of hierarchy is important and the effects of perturbing individual components are measured in a genome-wide and quantitative manner. When applied to the glucose regulatory system (Figure 5), it further supports the hypothesis that adjusting transcript levels of pathway members involved in storage carbohydrate metabolism is one of the most downstream transcriptional events. Although this concept is intuitive it has not, to our knowledge, been explicitly demonstrated before. While the synthase Tps1 is shown to be crucial for trehalose production [43], its transcription is little changed in deletion mutants of the glucose regulatory system (Figure 4). Based on the observed transcript changes, the phosphatase Tps2 is predicted to be the most downstream transcriptional component (Figure 5E; Figure 6) and the regulatory subunit Tsl1 is suggested to play an important role in communicating feedback (Figure 5E).

The network highlights the interplay between the mobilisation of glycogen, mediated by Gph1, and the biosynthesis of trehalose, mediated by Tps2 and Tsl1 (Figure 7). Recent studies suggest different roles for the storage carbohydrates glycogen and trehalose, where trehalose might be the preferred energy source for survival under a variety of conditions [44]. Consistently, our results show that transcript levels of Gph1, as well as Tps2 and Tsl1 are increased as the cell perceives low glucose concentrations through the deletion of a “high glucose” pathway member. Furthermore, a sequential relationship in the transcriptional changes upon the deletion of *GPH1* and *TPS2* is observed. Although based on transcriptional changes only, this suggests that glycogen is mobilised to replenish the internal glucose pool, whilst trehalose is built up. In addition, our results suggest that the ratio between these two processes is balanced by Tsl1 through feedback. Taken together, the results of this study imply that multiple inputs from different signalling pathways converge into the regulatory unit of Gph1, Tps2 and Tsl1 to balance the availability of storage carbohydrates and adjust the metabolic state of the cells accordingly.

## Conclusions

Pathways of the glucose regulatory system represent parallel pathways that are highly interconnected. Perceived alterations of external glucose levels lead to one main transcriptional response that varies in direction to mimic either a high or a low glucose response. Network analysis of the transcriptional changes suggests that this response is mediated by regulating storage carbohydrate biosynthesis, in particular by transcriptionally adjusting the abundance of Tps2 and Tsl1. An additional link to Gph1 possibly connects mobilisation of glycogen to trehalose biosynthesis to balance the availability of storage carbohydrates. This is an important aspect of the yeast glucose regulatory system and provides a basis for further studies to investigate the mechanistic and biochemical details.

## Methods

### Expression profiling and deletion strains

All experimental details of expression profiling the deletion mutants are provided in Additional file 3. In short, for expression profiling the deletion mutants, each mutant strain in the BY4742 background (Additional File 1) was profiled four times from two independently inoculated cultures and harvested in early mid-log phase in SC medium, supplemented with 2% glucose. Sets of mutants were grown alongside 56 WT cultures and processed in parallel. For expression profiling the glucose WT time-course, two overnight WT cultures were used to inoculate 50 ml cultures at an OD_600_ of 0.15. These were depleted of glucose by growing for 24 h and were used the next day to inoculate 500 ml cultures in fresh medium (SC, supplemented with 2% glucose) to an OD_600_ of 0.15. Samples for expression profiling were taken immediately after, as well as 3, 7.5, 15, 30, 60, 110, 150, and 300 minutes after inoculation into fresh medium.

Dual-channel 70-mer oligonucleotide arrays were employed with a common reference WT RNA. All steps after RNA isolation were automated using robotic liquid handlers. These procedures were first optimised for accuracy (correct FC) and precision (reproducible result), using spiked-in RNA calibration [45]. After quality control, normalisation, and dye-bias correction [46], statistical analysis was performed for each mutant versus a collection of 200 WT cultures. The reported FC is an average of the four replicate mutant gene expression profiles versus the average of all WTs. Transposable elements and mitochondrial genes were excluded from all analyses.

Incorrect strains from the deletion collections Euroscarf or Open Biosystems (15%) as indicated by aneuploidy (3%), incorrect deletion (7%), or additional spurious mutation affecting the gene expression profile (5%) were remade and re-profiled. Three strains were not available in either collection and thus made for this study (Additional file 1). None of the WT gene expression profiles had twelve or more genes changing compared to the average WT as determined by the same criteria as for the mutants (*p* < 0.01, FC > 1.7). This threshold was therefore applied to determine whether a mutant had a gene expression profile different from WT and was hence used for further analysis.

### Accession numbers

All microarray gene expression data is deposited in the public data repositories ArrayExpress (accession numbers E-TABM-1210 [deletion mutants] and E-TABM-1211 [glucose WT time-course] and GEO (accession number GSE33099 [deletion mutants and glucose WT time-course]. The data are also available as flat-file or in TreeView format from http://www.holstegelab.nl/publications/glucose_regulatory_system/.

### Construction of *tps2Δ* double mutants

*tps2Δ* MATa BY4741 strains were mated with *gpr1Δ* and *ram1Δ* MATα BY4742 strains and then sporulated. Double mutants were obtained from two independent spores through tetrad dissection.

### Construction of the glucose gene signature

The glucose gene signature is defined as the set of genes that is differentially regulated between the “high glucose” and “low glucose” pathway members. To this end, the following classification-like approach was used. Only deletion mutants with 30 or more transcripts changing significantly (*p* < 0.01, FC > 1.7) were used in the procedure (32 mutants). The dataset was randomly divided into a training (2/3 of the mutants, i.e. 21 mutants) and test (1/3 of the mutants, i.e. 11 mutants) set. Leave one out cross validation was applied on the training set to find all genes with a classification accuracy of 90% using a K-nearest neighbour (KNN) classifier with K equals three. In other words, genes were selected when correctly classifying 90% of all deletion mutants in the training set (19 out of 21 deletion mutants). This gene set was subsequently used to classify all mutants of the test set to obtain an independent estimate of the predictive power. This procedure was repeated 200 times. Genes were ranked according to their frequency of occurrence in these sets and the top *N* = 878 genes were selected, where *N* is the mean size of all 200 gene sets. For a schematic overview, see Supplementary Figure 3 in Additional file 3. Note that this classification-like approach was used in favour of a standard limma analysis to be able to select genes exhibiting only minor transcriptional changes but that still discriminate between the “high glucose” and “low glucose” pathway members.

### Functional enrichment analyses

For functional enrichment analyses, a hypergeometric testing procedure was performed using GO biological process annotations [24] as obtained from SGD [47] on September 3^rd^ 2011. The background population was set to 6,359 (the number of genes annotated in GO) and *p* values were Bonferroni corrected for multiple testing.

### Hierarchical network reconstruction

A directed network $G=\left(V,E\right)$ is constructed. Each vertex $v_{i}\in V$ represents a member of the glucose regulatory system and each edge $e_{x,y\;}\in E,E=\left\{L1,L2,F1,F2\right\}$ describes the relationship between two pathway members *x* and *y*. This relationship is defined as follows:

(1) $$e_{x,y}=\{\begin{array}{c} L1\text{,}\;d_{x,y}<0\;and\;c_{x,y\;}>0\\ L2\text{,}\;d_{x,y}>0\;and\;c_{x,y\;}<0\\ F1\text{,}\;d_{x,y}>0\;and\;c_{x,y\;}>0\\ F2\text{,}\;d_{x,y}<0\;and\;c_{x,y\;}<0 \end{array}$$

where *d*_*x,y*_ is the change of transcription of *y* upon the deletion of *x*

(2) $$d_{x,y}=\{\begin{array}{c} FC_{x,y}\;p_{x,y}<0.01\;and\;FC_{x,y}>1.5\;\\ \;0\text{,}\;else\text{,}\; \end{array}$$

and *c*_*x,y*_ is the significant cosine correlation between the gene expression profiles obtained upon the deletions of *x* and *y* respectively

(3) $$c_{x,y}=\{\begin{array}{c} c_{x,y}\;c_{x,y}<0\;or\;c_{x,y}>0.18\;\\ 0\;else\text{.} \end{array}$$

Significance of correlation is determined by a randomisation test as follows. First, transcript levels of each gene in a given gene expression profile are shuffled. Second, correlation between gene expression profiles is calculated for each mutant pair. This routine is repeated a 1,000 times to obtain a background distribution, and the lower 0.001 and upper 0.999 quantiles (corresponding correlation of 0 and 0.18) are applied as significance thresholds. Using these thresholds to determine significant correlation ensures that amongst 1,000 significant correlations lower than 0 or higher than 0.18 respectively, only one is likely to be random.

Robustness and stability of the resulting network was tested by varying the different parameters included. Application of pearson correlation instead of cosine correlation did not affect the resulting network. Small changes in strictness of *p* value and FC of transcription changes, or significance of correlation did affect the presence of individual edges in the network, but not the overall hierarchical structure and downstream position of Tsl1 and Tps2.

## Abbreviations

cAMP: Cyclic AMP; FC: Fold-change; GO: GeneOntology; KNN: K-nearest neighbour; SC: Synthetic Complete medium; WT: Wildtype.

## Competing interest

The authors declare that they have no competing interests.

## Authors’ contributions

EA planned and carried out experiments of deletion mutants, analysed the data, and wrote the manuscript. KS planned and carried out bioinformatics analyses, analysed the data and wrote the manuscript. TM and NB planned and carried out the glucose WT time-course experiment. LvdP contributed deletion mutant data. LB supported bioinformatics analyses. DvL technically assisted DNA microarray experiments. FH conceived the study and revised the manuscript. PK conceived the study and wrote the manuscript. All authors read and approved the final manuscript.

## Supplementary Material

Additional file 1 **Strains used in this study.** This Excel file contains information on all strains used in this study. Click here for file

Additional file 2 **Categorisation of components of the glucose regulatory system.** This Excel file contains literature- and data-based categorisation of all glucose signalling pathway members. Click here for file

Additional file 3 **Supplementary Figure **1-3**and Supplementary Experimental Procedures.** This PDF file contains three additional figures in support of the main text, as well as extensive descriptions of experimental procedures used in this study [48-56]. Click here for file

Additional file 4 **Supplementary analysis of the glucose signature.** This Excel file contains all genes included in the glucose gene signature, GO enrichments of the glucose gene signature, and GO enrichments of transcriptionally changed genes per deletion mutant excluding the glucose signature. Click here for file
