# Supplementary material for: Yeast glucose pathways converge on the transcriptional regulation of trehalose biosynthesis
Source: BMC Genomics. 2012 Jun 14;13:239. doi: 10.1186/1471-2164-13-239 (PMC3472246; doi:10.1186/1471-2164-13-239)
Supplement: Additional file 3 — Supplementary Figure 1-3and Supplementary Experimental Procedures. This PDF file contains three additional figures in support of the main text, as well as extensive descriptions of experimental procedures used in this study [48-56]. [file 1471-2164-13-239-S3.pdf]

Supplementary Information for

Yeast glucose pathways converge on the transcriptional regulation of trehalose biosynthesis

Eva Apweiler[1,2], Katrin Sameith[1,2], Thanasis Margaritis[2], Nathalie Brabers[2], Loes van de Pasch[2], Linda V. Bakker[2,3], Dik van Leenen[2], Frank C.P. Holstege[2], Patrick Kemmeren[2]

[1]Equal contribution

[2]Molecular Cancer Research, University Medical Centre Utrecht, Universiteitsweg 100, Utrecht, the Netherlands

[3]Netherlands Bioinformatics Centre, Geert Grooteplein 28, 6525 GA, Nijmegen, the Netherlands

Table of Contents

Supplementary Figure 1 3
Supplementary Figure 2 4
Supplementary Figure 3 5
Supplementary Experimental Procedures 6
Data availability 6
Yeast Strains 6
Quality control on strains from the deletion collection 6
Yeast growth for expression profiling 7
Yeast growth for expression profiling in Erlenmeyers (1) 7
Yeast growth for expression profiling in Tecan platereader (2) 8
Yeast growth for expression profiling the glucose WT time-course 8
Experimental design 9
DNA microarrays 9
RNA extraction 9
RNA purification for growth in Erlenmeyers 10
RNA purification for growth in Tecan platereader 11
External controls 11
RNA amplification and labelling 12
Hybridisation 13
Scanning and image analysis 14
Microarray quality control 14
Data normalisation 16
Statistical analysis of gene expression profiles 16
References 17

Supplementary Figure 1

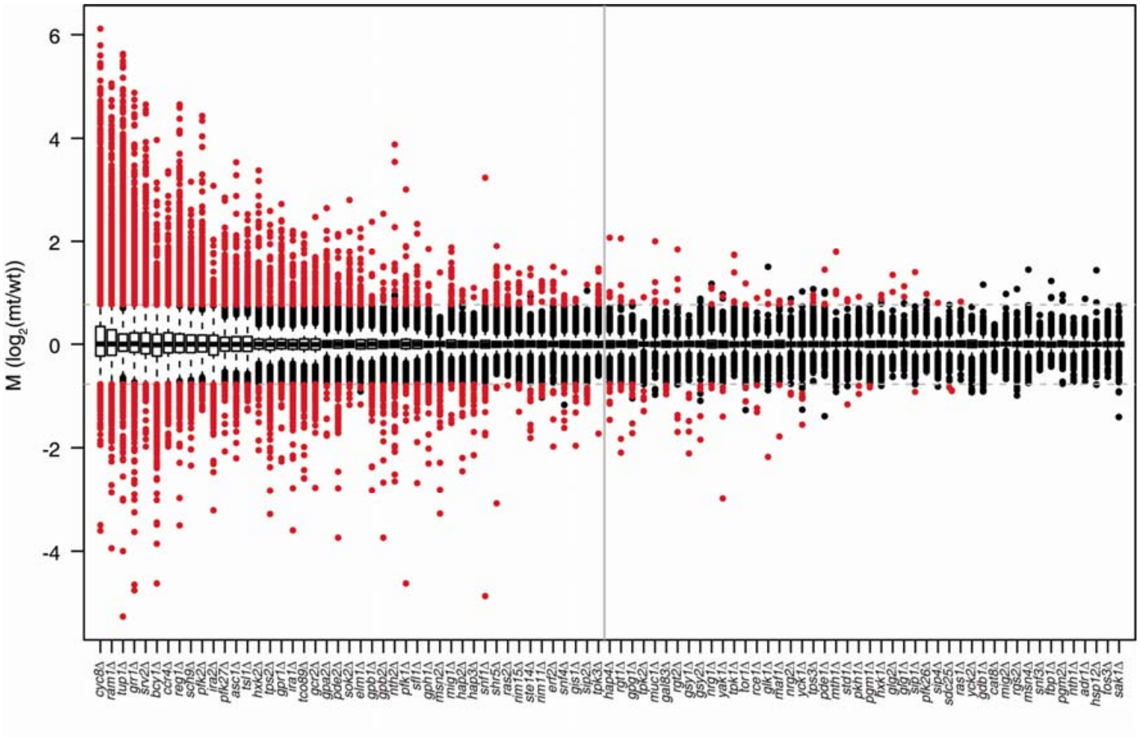

Activity profiles of all deletion strains

Activity profiles are ranked as box-whisker plots showing fold changes (vertical axis) with significantly changing genes ($p < 0.01$, FC > 1.7) as red dots and unresponsive genes as black dots. Dashed grey lines indicate 1.7-fold change. The solid grey line is the threshold for distinguishing deletions with significant gene expression profiles (≥ 12 genes changing) versus deletions that behave similar to WT (< 12 genes changing). This threshold is based on the maximum number of transcript changes observed in the 56 WT gene expression profiles, excluding mitochondrial genes and transposable element genes.

Supplementary Figure 2

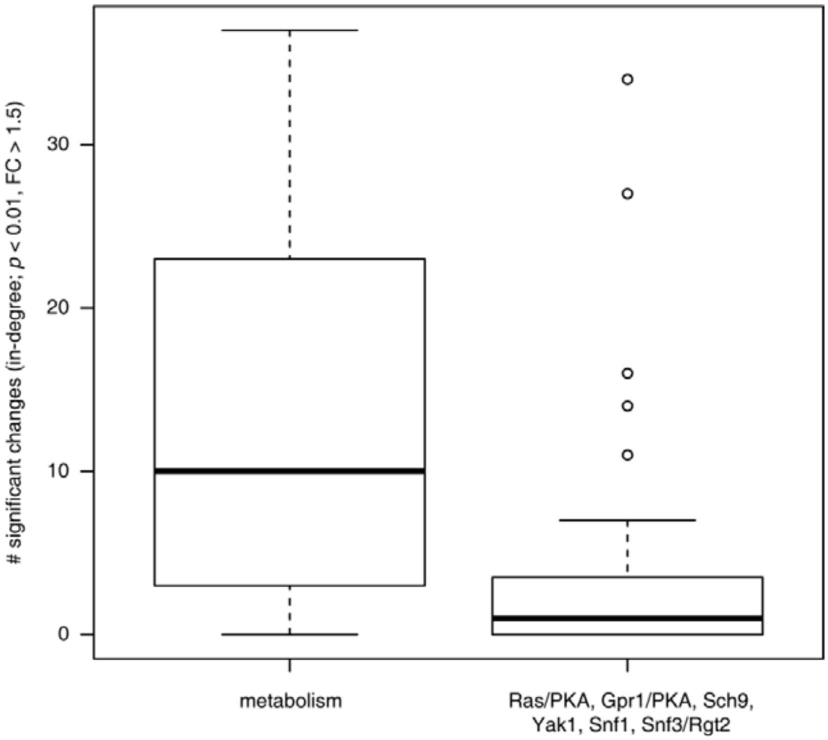

Transcription of metabolic pathway members significantly changes more frequently than transcription of signalling pathway members

Box-whisker plots depicting the number of mutants in which the transcription of members of the metabolic pathways (left) and the six signalling pathways (right) is significantly changed ($p < 0.01$ and FC > 1.5). Both distributions significantly differ from each other (Wilcoxon rank-sum test, $p = 6.0E-6$).

Supplementary Figure 3

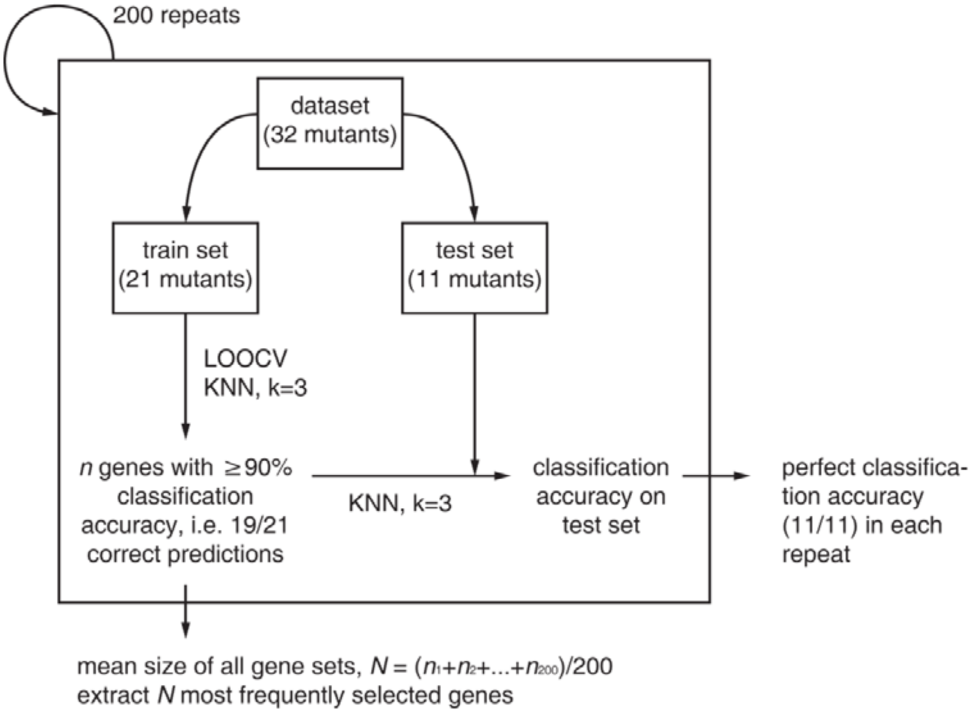

Schematic overview of the classification like procedure used to derive the glucose gene signature

Supplementary Experimental Procedures

All protocols related to DNA microarray expression profiling are also available from ArrayExpress (http://www.ebi.ac.uk/microarray) [1] with the accession numbers as indicated in the relevant sections.

Data availability

Raw data and normalised gene expression profiles are available from ArrayExpress (accession numbers E-TABM-1210 [deletion mutants]; E-TABM-1211 [glucose WT time-course]) and from GEO (accession number GSE33099 [deletion mutants and glucose WT time-course]). Expression ratios (log_2 FC) and *p* values of all profiles are also available as text-files (del_mutants_data.txt; time-course_data.txt) that can be downloaded from the supporting website (http://www.holstegelab.nl/publications/glucose_regulatory_system/). The expression changes on individual genes can also be viewed after installing Java Treeview [2] (http://jtreeview.sourceforge.net/), downloading the zipped TreeView files (del_mutants_treeview.zip; time-course_treeview.zip) available from the supporting website, extracting the files and opening the .cdt file. When viewing the data, individual strains and transcripts can be found quickly using the “Find” function.

Yeast Strains

All strains are isogenic to S288c, BY4742 [3]. The selection of glucose signaling components was manually curated from literature, resulting in 91 viable deletion strains. Haploid MATα gene deletion strains were initially obtained as two separate copies from the deletion collections Euroscarf (Frankfurt, Germany) or Open Biosystems (Huntsville, USA). Different problems, detailed below, were encountered for 16 strains in the collection. These were re-made. New deletion mutants were constructed using the kanamycin cassette from pFA6a-kanMX6 [4]. All strains used are described in Supplementary Table 1.

Quality control on strains from the deletion collection

In 16 strains from the collection, the gene expression profiles revealed different defects, annotated in the strain list in Supplementary Table 1. Note that such defects may be common to all copies of the collection but could also have arisen due to our handling of these strains. All these strains were re-made. Three different types of defects were encountered and are described below. All 91 strains selected for expression profiling passed our quality control criteria.

Incorrect deletion

In some deletion strains the supposed deleted gene was not obviously downregulated. This is usually due to already low expression in WT. In all such cases the deletion strain was checked by two PCRs, one using two primers outside the presumed deleted gene and another using one primer outside the gene and the other primer in the middle of the marker. PCR reactions were positive for four strains and the gene expression profiles were kept. Six deletion strains did not have the desired gene deleted and were subsequently re-made and re-profiled.

Aneuploidy

Aneuploidy is revealed in the gene expression profiles by analysis of expression changes in the context of chromosome location, one of the standard quality controls (QCs) performed on all gene expression profiles. Expression profiling revealed aneuploidy in three deletion mutants in the collection, sometimes as part of a chromosome, sometimes with one or more complete chromosomes involved. Aneuploid mutants were re-made and re-profiled.

Spurious mutations

Four deletion strains passed the QC criteria of correct deletion and no aneuploidy but had surprising gene expression profiles in light of what was previously known about the knockout gene. These were re-made and re-profiled.

Yeast growth for expression profiling

During this study the protocol for yeast growth was optimized to grow more mutants at the same day in a less labour intensive way. Two slightly different protocols for yeast growth were thus used in this study. These protocols only differed in culture volume and equipment. Detailed comparative analysis of the same mutants grown with both protocols revealed no difference in expression. Both protocols are described below. For strains grown in the Tecan platereader, an automated method for RNA purification was used.

Yeast growth for expression profiling in Erlenmeyers (1) (ArrayExpress accession PUMCU-36)

Strains were streaked from -80°C stocks onto plates and grown for 3-5 days depending on growth rate. Liquid cultures were inoculated with independent colonies and grown overnight in Synthetic Complete (SC) medium: 2gr/l Drop out mix Complete and 6.71gr/l Yeast Nitrogen Base without AA, Carbohydrate & w/AS (YNB) from US Biologicals (Swampscott,

USA) with 2% D-glucose. Overnight cultures were diluted to an OD600 of 0.15 in 60 ml fresh medium and grown at 30°C, 230 rpm shaking incubator, 250 ml Erlenmeyer flasks. Growth curves were made for the mutant cultures (two cultures from two isolates) as well as for two WT inoculates, grown in parallel. Mutant and WT cells were harvested by centrifugation (4000 rpm, 3 min) at mid-log phase at an OD600 of 0.6, and pellets were immediately frozen in liquid nitrogen after removal of supernatant. No more than four cultures were harvested simultaneously to decrease processing time. Note that OD600 measurements of cells are spectrophotometer dependent. OD600 0.6 (±0.1) corresponds to early mid-log phase for these cultures. It is essential to harvest at an OD600 that corresponds to early mid-log phase for WT cultures. High resolution time-course analysis of the entire growth curve showed that early to mid-log phase represents the window during which WT gene expression profiles are identical along the growth curve. WT cultures start showing significant changes in gene expression (after mid-log phase) long before growth slows down (see also [5]). Adherence to a strict early mid-log OD600 of 0.6 window for harvesting is particularly important for (a minority of) mutants that have significantly slower growth compared to WT, since for some of these mutants, OD600 of 0.6 is further along their relative growth curve. Problems associated with this are overcome if all mutants are harvested at an OD600 that represents early mid-log for WT cultures.

Yeast growth for expression profiling in Tecan platereader (2) (ArrayExpress accession P-UMCU-50)

Strains were streaked from -80°C stocks onto plates and grown for 3-5 days depending on growth rate. Liquid cultures were inoculated with independent colonies and grown overnight in Synthetic Complete (SC) medium: 2gr/l Drop out mix Complete and 6.71gr/l Yeast Nitrogen Base without AA, Carbohydrate & w/AS (YNB) from US Biologicals (Swampscott, USA) with 2% D-glucose. Overnight cultures were diluted to an OD600 of 0.15 in 1.5 ml fresh medium and grown at 30°C in a 24 well plate in a Tecan Infinite F200 under continuous shaking. Growth curves were made for the mutant cultures (two cultures from two isolates) as well as for two WT inoculates, grown in parallel. Mutant and WT cells were harvested by centrifugation (6100 rpm, 3 min) at mid-log phase at an OD600 of 0.6, and pellets were immediately frozen in liquid nitrogen after removal of supernatant.

Yeast growth for expression profiling the glucose WT time-course (ArrayExpress accession P-UMCU-47)

For expression profiling the glucose WT time-course, two overnight WT cultures were used to inoculate 50 ml cultures at an OD_{600} of 0.15. These were depleted of glucose by growing

for 24 hours and were used the next day to inoculate 500 ml cultures in fresh medium to an OD_{600} of 0.15. Samples for expression profiling were taken immediately after, as well as 3, 7.5, 15, 30, 60, 110, 150, and 300 minutes after inoculation into fresh medium.

Experimental design

Two channel microarrays were used. RNA isolated from a large amount of WT yeast from a set of cultures was used as a common reference. This common reference was used in one of the channels for each hybridisation and used in the statistical analysis to obtain an average gene expression profile for each deletion mutant relative to the WT. Two independent cultures were hybridised on two separate microarrays. For the first hybridisation the Cy5 (red) labelled cRNA from the deletion mutant is hybridised together with the Cy3 (green) labelled cRNA from the common reference. For the replicate hybridisation, the labels are swapped. Each gene is represented twice on the microarray, resulting in four measurements per mutant. Using the Erlenmeyer growth protocol up to five deletion strains were grown on a single day. In the Tecan platereader, up to eleven deletion strains could be grown on a single day. WT cultures were grown parallel to the deletion mutants to assess day-to-day variance. For the glucose WT time-course, WTs harvested at different time points were compared against time point zero.

DNA microarrays (ArrayExpress accession A-UMCU-10)

DNA microarray slides containing 70-mer oligonucleotides from the Operon® Array-Ready Oligo Set v1.1 (Operon biotechnologies, Huntsville, USA) were printed using the following protocol (ArrayExpress accession P-UMCU-34). Oligos are resuspended and adjusted to 10 µM in 150 mM phosphate buffer, pH8.5 in Genetix 384-wells plates. Array production is done using a Biorobotics MicroGrid II spotter located in a dust-free, temperature- and humidity-controlled cleanroom (ISO7/downflow ISO5), 18°C, 48% humidity. Slide (CodeLink Activated Slides, SurModics, USA) postprocessing is carried out according to the following CodeLink protocol. Printed slides are kept at 20°C, 75% humidity for 24 hours. Residual reactive groups are blocked using pre-warmed blocking solution (50 mM ethanolamine, 0.1 M Tris (pH 9) at 50°C for 30 min; slides are rinsed thoroughly with milliQ water; slides are washed with 4x SSC, 0.1% SDS (pre-warmed to 50°C) for 30 min at 50°C on a shaker. Slides are rinsed with milliQ water, dry-centrifuged and stored desiccated at room temperature. Each gene probe is spotted twice on the array, which contain an additional 2838 control features for external control normalisation and quality control [6].

RNA extraction (ArrayExpress accession P-UMCU-37 and P-UMCU-51).

Total RNA was prepared by phenol extraction and cleaned up using Qiagen’s RNeasy kit or on a customized Sciclone ALH 3000 Workstation. Frozen cells (-80°C) were resuspended in 500 µl Acid Phenol Chloroform (Sigma, 5:1, pH 4.7). Immediately an equal volume of TES-buffer (TES: 10 mM Tris pH 7.5, 10 mM EDTA, 0.5% SDS) was added. Samples were vortexed very hard for 20 seconds and incubated in a water bath for 10 minutes at 65°C and vortexed again. Samples were placed in a thermomixer (65°C, 1400 rpm) for 50 minutes. Samples were spun down for 20 minutes at 14000 rpm at 4°C. Phenol extraction was repeated once, followed by a Chloroform:Isoamyl-alcohol (25:1) extraction. RNA was precipitated with Sodium Acetate (NaAc 3M, pH 5.2) and ethanol (96%, -20°C). Pellet was washed with Ethanol and dissolved in sterile water (MQ), snapfrozen and stored at -80°C.

RNA purification for growth in Erlenmeyers (ArrayExpress accession P-UMCU-37)

RNA purification was performed using Qiagen's RNAeasy kit using the following protocol.

- Take 50 µg total RNA
- Adjust volume to 300 µl with MQ
- Add 160 µl RLT buffer
- Add 240 µl 96% ethanol, mix by pipetting and directly apply to the RNAeasy column
- Centrifuge 8000 rpm 15 sec
- Add 350 µl buffer RW1
- Centrifuge 8000 rpm 15 sec
- Add 10 µl DNase to 70 µl RDD buffer and apply to the column, incubate 15 min at room temperature
- Add 350 µl buffer RW1
- Centrifuge 8000 rpm 15 sec
- Add 500 µl RPE buffer
- Wait 1 minute and centrifuge 8000 rpm 15 sec
- Add 500 µl RPE buffer
- Wait 1 minute and centrifuge 8000 rpm 15 sec
- Discard flow-through and centrifuge 10,000 rpm 2 min
- Transfer column to a 1.5 ml tube
- Add 35 µl MQ
- Wait for 4min and centrifuge 10,000 rpm 1 min
- Add the same 35 µl MQ
- Wait for 4min and centrifuge 10,000 rpm 1 min

- Transfer eluate to a new tube as some column material may stick to the bottom of your tube
- Measure concentration (dilute 50 times (4 µl in 196 µl)) + bioanalyser (50 ng)
- Directly make 1 aliquot of 30 µl with a concentration of 0.6 µg/µl
- Measure the concentration of this aliquot again (4 µl in 96 µl MQ); must be 0.6 µg/µl.
- Adjust or remake if necessary
- Split into 2 aliquots of > 10 µl each
- Freeze in liquid N2 and store at -80°C

RNA purification for growth in Tecan platereader (ArrayExpress accession P-UMCU-51)

Following protocols were automatically performed on a customized Sciclone ALH 3000 Workstation (Caliper LifeSciences) that included a PCR PTC-200 (Bio-Rad Laboratories), SpectraMax 190 spectrophotometer (Molecular Devices), and a magnetic bead-locator (Beckman).

RNA clean-up protocol description:

- Concentration of all RNAs is measured by diluting 5 µl RNA on the SpectraMax
- 12.5 µl DNaseI dilution (RNase-free DNAse kit, Qiagen, nr 79254, diluted 1:5 in RDD) is mixed with remaining 87.5 µl RNA-solution and incubated at 18°C for 15 minutes
- RNA is purified and concentrated with RNAClean (Agencourt, Beckman) according to manufacturer's protocol, to an end volume of 25 µl
- 4 µl cleaned RNA is diluted and measured on the SpectraMAX, concentrations are normalised to 0.2 µg/µl in each well
- 5 µl of cleaned and normalised RNA is used to set up a startplate for RNA amplification.
- 1 µl of cleaned and normalised RNA is used to check integrity by running on a QiaXcel system.
- All plates are snap-frozen and stored at -80°C until further use.

External controls

External control poly-A+ RNAs were added in equimolar amounts to the total RNA to enable monitoring of global changes in mutants [6]. Constructs containing *Bacillus subtilis* genes (*ycxA*, *yceG*, *ybdO*, *ybbR*, *ybaS*, *ybaF*, *ybaC*, *yacK* and *yabQ*) cloned between the *Xba*I and

*Bam*HI sites in pT7T3 (Amersham Pharmacia Biotech) were made, with an additional 30-nucleotide poly(A) sequence between the gene and the *Xho*I site. For making RNA, plasmids were digested with *Xho*I for use in *in vitro* transcription reactions using MEGAscript-T7 (Ambion).

RNA amplification and labelling (ArrayExpress accession P-UMCU-38)

All RNA amplification and labelling procedures are performed in 96 wells plates (4titude, Bioke) on a customized Sciclone ALH 3000 Workstation (Caliper LifeSciences) that included a PCR PTC-200 (Bio-Rad Laboratories), SpectraMax 190 spectrophotometer (Molecular Devices), and a magnetic bead-locator (Beckman).

RNA amplification

For RNA amplification, total RNA samples were diluted to 0.6 µg/µl (growth in Erlenmeyers) or 0.2 µg/µl (growth in Tecan platereader) and 5 µl is put in a 96-wells plate (Abgene).

All subsequent steps were performed by the following robot script:

- Mix1 containing 100 ng T7 Mlu VN primer (custom mix) and external control RNA per 5 µl is added and mixed in each well. Plate is incubated at 70°C for 10 minutes and cooled to 48°C.
- Mix2 containing 4 µl 5x 1st strand buffer, 2 µl 0.1 M DTT (Invitrogen), 1 µl RNAse Inhibitor (Boehringer), 1 µl 20 mM dNTPs (GE Healthcare), 1 µl linear acrylamide, and 1 µl SuperScriptIII (Invitrogen) per sample is pre-warmed to 48°C; 10 µl per sample is added and mixed in each well.
- Plate is incubated at 48°C for 2 hours and cooled to room temperature.
- 106 µl water and subsequently mix3 containing 15 µl second strand buffer, 3 µl 20mM dNTPs (GE Healthcare), 1 µl T4 DNA ligase, 4 µl E.coli DNA polymerase I and 1µl RNAseH (Promega) is added and mixed in each well.
- Plate is incubated at 16°C for 2 hours, at 65°C for 10 minutes. ds cDNA product is purified and concentrated with RNAClean (Agencourt, GC biotech) according to manufacturers' protocol, to an end volume of 25 µl.
- 8 µl cDNA is put in a 96 wells plate and mixed with 12 µl IVT mix containing 2 µl 10x rxn-buffer, 2 µl of each ATP, CTP, GTP, 0.6 µl UTP, 2 µl T7 enzyme mix (MegaScript kit, Ambion, Applied Biosystems), and 2.1 µl 50 mM 5-(3-aminoallyl)-UTP (Ambion, Applied Biosystems).

- Plate is incubated at 37°C for 4 hours. cRNA product is purified with RNAClean (Agencourt, Beckman) according to manufacturer's protocol.
- Concentration is measured (SpectraMax 190) and adjusted to 600 ng/µl. Resulting cRNA plates are sampled for Bioanalyzer QC, snapfrozen and stored at -80°C.

Labelling

- NHS-ester Cy3 or Cy5 dye (Amersham PA 23001 and 25001): entire tube is resuspended in 100 µl DMSO (Merck 8.02912.10).
- 8 µl of each cRNA sample (0.6 µg/µl) is put in a 96-wells plate (Abgene), the accompanying reference sample is put in the next column (final step combines Cy3 and Cy5 labelled material).
- 3 µl 0.5 M Sodium Bicarbonate buffer, pH 9 is added and mixed to all wells.
- 3 µl Cy-dye solution is added and mixed to the appropriate wells, plate is incubated at 18°C for 1 hour.
- 4.5 µl 5 M hydroxylamine is added and mixed, incubated at 18°C for 15 minutes. Labelled cRNA product is purified with RNA Clean (Agencourt, GC biotech) according to manufacturers' protocol.
- RNA concentration and labelling incorporation are measured (SpectraMax 190).
- 2.5 µg of each labelled sample and reference cRNA are pooled and subjected to fragmentation according to protocol (Ambion, Applied Biosystems), 15 minutes at 70°C. Samples are stored at -20°C until hybridisation.

Hybridisation (ArrayExpress accession P-UMCU-39)

2.5 µg of each labelled sample in a total volume of 60 µl is combined with 60 µl 2xhybmix, containing 50% formamide, 10xSSC, 0.2% SDS, 200 µg/ml herring sperm DNA. The hybridisations were performed for 16 hours at 42°C in a HS4800Pro hybstation (Tecan, Männedorf, Switzerland) as detailed below. Hybridisation and scanning were performed in a temperature- and humidity-controlled laboratory in a low ozone atmosphere.

- 60 µl labelled sample is combined with 60 µl 2x-hybmix, containing 50% formamide, 10xSSC, 0.2% SDS, 200 µg/ml herring sperm DNA
- Hybridisations of spotted oligo-arrays (Codelink glass) are performed on an HS4800Pro Hybstation (Tecan, Männedorf, Switzerland).
- Priming: 5xSSC, 0.1%SDS.
- Probe injection: pre-hyb, 5xSSC, 25% formamide, 0.1%SDS, 1%BSA, total volume 110 µl.

- Hybridisation: 45 min at 42°C.
- Wash 2x: milliQ.Wash: 5xSSC, 0.1%SDS.
- Probe injection: sample. Volume 110 µl.
- Hybridisation: 16 hours at 42°C.
- Wash 2x: 1xSSC, 0.2%SDS at 23°C.
- Wash 2x: 0.1xSSC, 0.2%SDS at 23°C.
- Wash 2x: 0.1xSSC at 23°C.
- Drying: blow with nitrogen for 3 minutes at 30°C.

Scanning and image analysis

Slides were scanned using a G2565AA scanner (Agilent, California, USA) at 100% laser power and 30% PMT (ArrayExpress accession P-UMCU-40). After scanning, the intensities for the Cy5 (Red) and Cy3 (Green) channels were automatically extracted using the batch-processing module in ImaGene 8.0.1 (Biodiscovery, California, USA) (ArrayExpress accession P-UMCU-42). For spot finding a local flexibility of 2.0 pixels was used. For segmentation the following settings were used: background buffer: 3.0; background width: 3.0; signal percentages: 3% (low), 97% (high); background percentages: 3% (low), 97% (high). Measurements exported: mean, median, total, standard deviation and area of the foreground and background signals. Batch editor configuration files are available upon request.

Microarray quality control

Each hybridisation performed within this project was subjected to a number of quality controls. Some of these are based on the data from one single hybridisation, while others are based on comparing data from one single hybridisation against the WT grown in parallel.

Quality controls for a single hybridisation

For each hybridisation a quality report was generated that contained a number of quality controls. For all of these quality controls either raw non-background corrected mean intensity values were used, or data normalised on all gene probes using the print-tip LOESS [7] algorithm (*marray* R package version 1.20.0) with a window span of 0.4 and excluding genes with nearly saturated signals (i.e. mean intensity > 215) for the loess curve estimation was used. The following plots are generated:

- Spatial distribution plot of all Signal-to-Noise ratios (SNR) according to the position on the microarray for the red and green raw intensity values. SNRs are binned

according to the number of standard deviations that the mean intensity signal is above the background and plotted using different colours; SNR < 2: black; 2 ≤ SNR ≤ 3: red; 3 ≤ SNR < 4: orange; SNR ≥ 4: yellow.

- Spatial distribution plot of all *M* (log2(*R*/*G*)) ratios according to the position on the microarray for both raw and normalised data.
- MA plot (log2(*R*/*G*) vs. 0.5*log2(*R***G*)) for both raw and normalised data. For each data type three different MA plots were generated, one containing all probes present on the microarray, one containing all gene probes present on the microarray and one containing all quality controls and external control probes present on the microarray.
- Spatial distribution plot of local background intensity values according to the position on the microarray for the red and green channels. Local background intensity values are binned relative to the average background intensity of the entire microarray; background > 2.1*average: black; 1.7*average < background ≤ 2.1*average: red; 1.3*average < background ≤ 1.7*average: orange; background < 1.3*average: yellow.
- Histograms of the distribution of raw red and green intensities and raw *M* ratios.
- Chromosomal location plot of all normalised *M* ratios according the chromosomal position of the gene represented by the probe on the microarray.
- Histograms of the distribution of normalised *M* ratios per chromosome. Individual histograms are coloured red if the chromosome-specific *M* is significantly larger than the average *M* over all chromosomes by at least 0.15; green if they are similarly smaller and yellow otherwise. Significant in this case means an average Benjamini-Hochberg FDR corrected *p* value of < 10-6. In addition to the visual quality controls described above, a number of numerical quality measures were calculated for each hybridisation as proposed by [8]. Each probe present on the microarray was classified as good, marginal or bad depending on the calculated quality measures for the raw intensity values. Good spots have the following characteristics: SNR ≥ 4; background flatness ≥ 6; signal consistency ≥ 1.1; foreground signal > background signal. Bad spots have the following characteristics: SNR ≤ 2; background flatness ≤ 4; signal consistency ≤ 0.9; foreground signal ≤ background signal (black hole). Background flatness is calculated by comparing the background intensity of a probe to the mean background intensity of the sub grid. Signal consistency is calculated from the signal coefficient of variation. An overall percentage of good marginal and bad spots for the gene and control probe groups is reported and judged (also relative to other hybridisations within the project, see Quality controls for entire project).

Quality controls for the entire project

To assess the performance of individual hybridisations within this project, hybridisations were ranked according to the percentage of good gene probes. Individual hybridisations for deletion mutants with fewer than 93% good gene probes where considered outliers and were removed. Each deletion mutant gene expression profile was compared against the gene expression profile of the WT grown in parallel to ensure that the effect that we observe in the deletion mutant is specific for the deletion mutant and is not present in the WT grown in parallel. If a significant overlap was found between the significantly regulated genes in the deletion mutant and the WT grown in parallel as determined by a hypergeometric test ($p < 0.01$), the hybridisations for the deletion mutant were removed. Using the quality controls mentioned, individual hybridisations for the deletion mutants were assessed for their data quality. If a hybridisation showed an irregular pattern in any of the visual quality control plots due to for instance air bubbles, scratches on the surface, dust, minor printing defects, uneven spread of the hybridisation solution or low RNA amounts; had a relatively low percentage of good spots or had a significant overlap with the WT grown in parallel, the hybridisation was excluded from further analysis and the RNA for the deletion mutant was re-hybridised if it seemed like the deletion mutant would result in a significant gene expression profile.

Data normalisation (ArrayExpress accession P-UMCU-43)

Microarray data normalisation was performed on mean intensity values using print-tip LOESS as described in (Yang et al., 2002) and implemented in the *marray* R package version 1.20.0, using no background subtraction and a window span of 0.4. Probes flagged as absent, or with a (nearly) saturated signal (*i.e.* $> 2^{15}$) in either channel were not considered for the estimation of the LOESS curve. Gene-specific dye bias (GSDB) is not addressed by LOESS, and was corrected only in the deletion mutant dataset using Gene-And Slide-Specific Correction (GASSCO) [9] implemented in the R package *dyebias* version 1.4.3. The total GSDB is expressed as the product of two factors: an intrinsic gene specific dye bias (*iGSDB*), and the slide bias *F*. The *iGSDB* is estimated as the average *M* (*i.e.* log2(Cy5/*Cy*3)) in a set of 200 WT vs. WT hybridisations (ArrayExpress accession E-TABM-773). The *iGSDB*s are estimated only once for one set of probes and protocols, and were used for all slides. The slide bias *F* was estimated for each hybridisation separately, using two groups of probes, having the strongest red or green biases, defined as those with an *iGSDB* in the top and bottom 5th percentiles of *iGSDB*s, respectively. The slide bias is the mean of the red and green probe group's median (*M* / *iGSDB*)-ratio.

Statistical analysis of gene expression profiles

For each deletion mutant the replicate hybridisations from two independent cultures were compared to the WT cultures grown on the same day in parallel and a pool of 200 WT replicates [10] grown throughout the project through a common reference. Due to a change in the shaker used for yeast growth during this project, two different WT pools were constructed. *P* values were obtained from the *limma* R package version 2.12.0 [11] after Benjamini-Hochberg FDR correction. Genes were considered significantly changed when the fold change (FC) was > 1.7 and the p value < 0.01. Transposable elements and mitochondrial genes were excluded from further downstream analysis, as well as YDL196W, since it is frequently upregulated indicating that expression of this gene was downregulated in the common reference WT culture, likely due to a spurious mutation. For determining which deletion mutants have a significant effect on mRNA expression levels, all deletion mutants and the 56 WT cultures grown in parallel were ranked by the number of significantly changing genes. No WT had twelve or more genes changing and based on this the deletion mutants were classified into two groups: profiling (≥ 12 genes changing) and non-profiling (< 12 genes changing). R (template) scripts for running the *limma* statistical analysis are available upon request.

For the glucose WT time-course, replicate hybridisations from two independent WT cultures at different time points were compared to the same cultures at time point 0 using the *limma* R package as described above. As for the deletion mutant dataset, transposable elements, mitochondrial genes, and YDL196W were excluded.

References

1. Parkinson H, Sarkans U, Shojatalab M, Abeygunawardena N, Contrino S, Coulson R, Farne A, Lara GG, Holloway E, Kapushesky M, Lilja P, Mukherjee G, Oezcimen A, Rayner T, Rocca-Serra P, Sharma A, Sansone S, Brazma A: **ArrayExpress--a public repository for microarray gene expression data at the EBI**. *Nucleic Acids Res* 2005, **33**:D553-555.

2. Saldanha AJ: **Java Treeview—extensible visualization of microarray data**. *Bioinformatics* 2004, **20**:3246 -3248.

3. Winzeler EA, Shoemaker DD, Astromoff A, Liang H, Anderson K, Andre B, Bangham R, Benito R, Boeke JD, Bussey H, Chu AM, Connelly C, Davis K, Dietrich F, Dow SW, El Bakkoury M, Foury F, Friend SH, Gentalen E, Giaever G, Hegemann JH, Jones T, Laub M, Liao H, Liebundguth N, Lockhart DJ, Lucau-Danila A, Lussier M, M'Rabet N, Menard P, et al.: **Functional Characterization of the S. cerevisiae Genome by Gene Deletion and Parallel Analysis**. *Science* 1999, **285**:901 -906.

4. Longtine MS, Mckenzie III A, Demarini DJ, Shah NG, Wach A, Brachat A, Philippsen P, Pringle JR: **Additional modules for versatile and economical PCR-based gene deletion and modification in Saccharomyces cerevisiae**. *Yeast* 1998, **14**:953-961.

5. Radonjic M, Andrau J-C, Lijnzaad P, Kemmeren P, Kockelkorn TTJP, van Leenen D, van Berkum NL, Holstege FCP: **Genome-Wide Analyses Reveal RNA Polymerase II Located Upstream of Genes Poised for Rapid Response upon S. cerevisiae Stationary Phase Exit**. *Molecular Cell* 2005, **18**:171-183.

6. van de Peppel J, Kemmeren P, van Bakel H, Radonjic M, van Leenen D, Holstege FCP: **Monitoring global messenger RNA changes in externally controlled microarray experiments**. *EMBO Rep* 2003, **4**:387-393.

7. Yang YH, Dudoit S, Luu P, Lin DM, Peng V, Ngai J, Speed TP: **Normalization for cDNA microarray data: a robust composite method addressing single and multiple slide systematic variation**. *Nucleic Acids Res* 2002, **30**:e15-e15.

8. Chen Y, Kamat V, Dougherty ER, Bittner ML, Meltzer PS, Trent JM: **Ratio statistics of gene expression levels and applications to microarray data analysis**. *Bioinformatics* 2002, **18**:1207 -1215.

9. Margaritis T, Lijnzaad P, van Leenen D, Bouwmeester D, Kemmeren P, van Hooff SR, Holstege FCP: **Adaptable gene-specific dye bias correction for two-channel DNA microarrays**. *Mol. Syst. Biol* 2009, **5**:266.

10. van Wageningen S, Kemmeren P, Lijnzaad P, Margaritis T, Benschop JJ, de Castro IJ, van Leenen D, Groot Koerkamp MJA, Ko CW, Miles AJ, Brabers N, Brok MO, Lenstra TL, Fiedler D, Fokkens L, Aldecoa R, Apweiler E, Taliadouros V, Sameith K, van de Pasch LAL, van Hooff SR, Bakker LV, Krogan NJ, Snel B, Holstege FCP: **Functional Overlap and Regulatory Links Shape Genetic Interactions between Signaling Pathways**. *Cell* 2010, **143**:991-1004.

11. Smyth GK, Michaud J, Scott HS: **Use of within-array replicate spots for assessing differential expression in microarray experiments**. *Bioinformatics* 2005, **21**:2067-2075.
